# Dietary Patterns, n-3 Fatty Acids Intake from Seafood and High Levels of Anxiety Symptoms during Pregnancy: Findings from the Avon Longitudinal Study of Parents and Children

**DOI:** 10.1371/journal.pone.0067671

**Published:** 2013-07-12

**Authors:** Juliana dos Santos Vaz, Gilberto Kac, Pauline Emmett, John M. Davis, Jean Golding, Joseph R. Hibbeln

**Affiliations:** 1 Faculty of Nutrition, Federal University of Pelotas, Pelotas, Rio Grande do Sul, Brazil; 2 Nutritional Epidemiology Observatory, Federal University of Rio de Janeiro, Rio de Janeiro, Rio de Janeiro, Brazil; 3 Centre for Child and Adolescent Health, School of Social and Community Medicine, University of Bristol, Bristol, United Kingdom; 4 Department of Psychiatry, University of Illinois at Chicago, Chicago, Illinois, United States of America; 5 National Institute on Alcoholism and Alcohol Abuse, National Institutes of Health, Bethesda, Maryland, United States of America; Wageningen University, Netherlands

## Abstract

**Background:**

Little is known about relationships between dietary patterns, n-3 polyunsaturated fatty acids (PUFA) intake and excessive anxiety during pregnancy.

**Objective:**

To examine whether dietary patterns and n-3 PUFA intake from seafood are associated with high levels of anxiety during pregnancy.

**Design:**

Pregnant women enrolled from 1991–1992 in ALSPAC (*n* 9,530). Dietary patterns were established from a food frequency questionnaire using principal component analysis. Total intake of n-3 PUFA (grams/week) from seafood was also examined. Symptoms of anxiety were measured at 32 weeks of gestation with the Crown-Crisp Experiential Index; scores ≥9 corresponding to the 85^th^ percentile was defined as high anxiety symptoms. Multivariate logistic regression models were used to estimate the OR and 95% CI, adjusted by socioeconomic and lifestyle variables.

**Results:**

Multivariate results showed that women in the highest tertile of the health-conscious (OR 0.77; 0.65–0.93) and the traditional (OR 0.84; 0.73–0.97) pattern scores were less likely to report high levels of anxiety symptoms. Women in the highest tertile of the vegetarian pattern score (OR 1.25; 1.08–1.44) were more likely to have high levels of anxiety, as well as those with no n-3 PUFA intake from seafood (OR 1.53; 1.25–1.87) when compared with those with intake of >1.5 grams/week.

**Conclusions:**

The present study provides evidence of a relationship between dietary patterns, fish intake or n-3 PUFA intake from seafood and symptoms of anxiety in pregnancy, and suggests that dietary interventions could be used to reduce high anxiety symptoms during pregnancy.

## Introduction

Excessive anxiety in pregnancy has been extensively investigated as a potential maternal risk factor for adverse outcomes for both mothers and their offspring [1]–[3]. Results from large cohort studies have associated gestational anxiety with low birth weight [4] and shorter length of gestation [5]. Additionally, clinical studies have provided evidence of the effect of higher anxiety in pregnancy and residual adverse effects on infant neurodevelopment, including stress regulation [6]–[8].

Anxiety has been extensively investigated, but little is known about nutritional variations that might be associated with a higher likelihood of occurrence of this outcome in pregnancy. Essential n-3 polyunsaturated fatty acids (PUFA) are nutritional components obtained exclusively from dietary sources, and fish and seafood are the major sources [9], [10]. This class of PUFA are involved in several brain functions, including regulation of mood [11], [12] and nutritional insufficiencies that may be implicated in susceptibility to mood disorders, such as depression and anxiety. The Avon Longitudinal Study of Parents and Children (ALSPAC) has previously provided evidence of a protective effect of fish consumption during pregnancy on symptoms of depression in pregnancy [13], and beneficial effects on child neurodevelopment, including higher verbal intelligence quotient [14] and early development of language and communication skills [15]. These benefits were assumed to be likely attributable to essential n–3 PUFA in fish and other seafood. However, other nutrients and dietary factors may also contribute to these benefits.

Epidemiological studies considering diet and common mental disorders have employed factor analysis to derive dietary patterns. Instead of looking at individual nutrients or foods, this method identifies patterns of food intake and examines its effect on prediction of disease risk [16]. Based on this approach, an Australian study with non-pregnant women found that a ‘traditional’ dietary pattern (characterized by vegetables, fruit, beef, lamb, fish and whole grain foods) was associated with reduced odds for bipolar disorder, while a ‘modern’ dietary pattern (characterized by fruits, salads, plus fish, tofu, beans, nuts, yogurt and red wine), was associated with increased likelihood of a bipolar disorder diagnosis [17]. In a Greek study, higher scores on a ‘healthy’ dietary pattern (characterized by vegetables, fruit, pulses, nuts, dairy products, fish and olive oil) during pregnancy were associated with reduced risk of postpartum depressive symptoms [18]. Because food habits vary among countries, the name attributed to a dietary pattern may be similar among studies, but differ in the type of food consumed; therefore direct comparisons are not possible.

To our knowledge nutritional benefits of dietary patterns and fish consumption in the UK have not been fully explored in relation to anxiety in pregnancy. ALSPAC had previously identified five dietary patterns in pregnancy. Thus, the aim of this study is to evaluate whether dietary pattern scores, frequency of seafood consumption and n-3 PUFA intake are associated with risk of high levels of anxiety symptoms in pregnancy. We hypothesized that low scores on health-conscious (characterized by salad, fruit, fruit juice, rice, pasta, oat/bran based breakfast cereal, fish, pulses, cheese and non-white bread) and/or traditional (characterized by vegetables, red meat and poultry) dietary patterns would be associated with high anxiety symptoms in pregnancy. Moreover, we expected that a lower intake of n-3 PUFA, consequence of a low frequency of seafood consumption, would be associated with greater risk of high levels of anxiety symptoms in pregnancy.

## Subjects and Methods

### Ethics statement

The ethical approval for the study was obtained from the Avon Longitudinal Study of Parents and Children (ALSPAC) Law and Ethics committee (IRB#00003312) and the local research ethics committees (Bristol and Weston, Southmead, and Frenchay Health Authorities). Written informed consent was obtained from all participants in the study.

### ALSPAC cohort study

ALSPAC was initiated to identify features of the environment that influence the health and development of children and their parents [19]. The initial population consisted of 14,541 pregnant women who resided in the Avon health authority area in southwest England and had an expected date of delivery between April 1991 and December 1992. More detailed information is available at (http://www.bristol.ac.uk/alspac). The representativeness of the ALSPAC cohort with respect to the population living in Avon county and the whole of Great Britain in 1991 has been assessed and is reported on the ALSPAC website (http://www.bristol.ac.uk/alspac/researchers/resources-available/cohort/represent/).

This manuscript uses the information collected on diet and anxiety from maternal self-completed questionnaire at 32 weeks' gestation. Women with multiple births (*n* = 199) were excluded from the present analysis. We only included the women for whom complete data were available on all confounding variables (Please, see *Statistical analysis* section). After exclusion for missing data, the final samples comprised 9,530 subjects (66% of the initial sample).

### Anxiety assessment

Maternal anxiety was measured using the eight anxiety items from the Crown-Crisp Experiential Index (CCEI), a validated self-rating inventory relating to free-floating anxiety, depression and somaticism [20]. The anxiety items were presented as follow: “do you feel upset for no obvious reason?”, “have you felt as though you might faint?”, “do you feel uneasy and restless?”, “do you sometimes feel panicky?”, “do you worry a lot?”, “do you feel strung-up inside?”, “do you ever have the feeling you are going to pieces?”, “do you have bad dreams which upset you when you wake up?” Each item had four possible categories in which the respondent indicated the frequency of symptoms as ‘never’, ‘not often’, ‘often’ or ‘very often’. In a pilot study of a random sample of 54 pregnant women attending a routine check-up, the Crown-Crisp anxiety subscale, correlated 0.70 and 0.76 with the State and Trait subscales of the Spielberger State-trait Anxiety Inventory respectively [21]. Internal consistencies (α) of the CCEI exceeded 0.80 for each of the four assessments. There is no established cut-off for this measure; we therefore identified as having high levels of anxiety symptoms women who scored in the top 15% (or as close to this as possible) at the 32 weeks antenatal assessment (a score of 9 or more on the 16-point scale).

### Dietary patterns and seafood exposure

Women completed a self-reported food frequency questionnaire at 32 weeks of gestation which comprised 110 questions enquiring about the frequency of consumption of 43 food groups and food items, and about daily consumption of a further eight basic foods [22]. The foods chosen were based on those used by Yarnell et al. (1983) [23] and modified in the light of a weighed dietary survey carried out in the study area [24]. Information on portion sizes is not relevant for the establishment of the patterns. Five dietary patterns in pregnancy have been previously identified from these data [25] using principal components analysis [16]: “Health-conscious”: salad, fruit, fruit juice, rice, pasta, oat/bran based breakfast cereal, fish, pulses, cheese, non-white bread; “Traditional”: vegetables, red meat, poultry; “Processed”: meat pies, sausages, burgers, fried foods, pizza, chips, white bread, eggs, baked beans; “Confectionery”: chocolate, sweets, biscuits, cakes, puddings, and “Vegetarian”: meat substitutes, pulses, nuts, herbal tea and high negative loadings for red meat and poultry. Dietary pattern scores were expressed in standard deviation units; each woman was represented in each of these five mutually independent scores.

Since seafood was the predominant source of essential n-3 PUFA in the diet of this population at this time, we estimated the total n-3 PUFA intake from seafood only, using the three questions that assessed seafood consumption: “How many times a week nowadays do you eat (a) white fish (cod, haddock, plaice, fish fingers, etc.), (b) dark or oily (tuna, sardines, pilchards, mackerel, herring, kippers, trout, salmon, etc.), or (c) shellfish (prawns, crabs, cockles, mussels, etc.)?” Each response had 5 predefined categories: never or rarely, once in 2 weeks, 1–3 times per week, 4–7 times per week, and more than once a day. These were converted to weekly frequencies of consumption (portions per week) of 0, 0.5, 2, 5.5, and 10, respectively. Portion sizes and type of fish were based on typical consumption patterns in UK and n-3 PUFA content was calculated using British food composition tables [26]. An estimation of energy intake was made using the food frequencies together with standard portion weights for each food.

### Potential confounders

To build the theoretical model for high levels of anxiety symptoms, the following independent variables were included in the analyses: Socio-demographic: age (<25, ≥25), education based on the highest educational qualification achieved [low: no more than a vocational qualification; medium: O-level (academic qualification normally taken at age 16****years) or equivalent; high: A-level (academic qualification at 18 years) or higher)]; Socio-economic: work status (employed, unemployed), housing tenure [mortgaged/owned, council rented (public housing), other]; crowding, persons per room (<1; ≥1); Lifestyle: maternal smoking during first trimester of pregnancy (yes, no), maternal alcohol consumption during first trimester of pregnancy (yes, no); Obstetric: parity (number of previous pregnancies resulting in a live birth or a late fetal death: 0, 1, ≥2), previous history of abortion (yes, no); previous history of miscarriage (yes, no); Chronic stress and life events: the mother's life events in childhood scale (≤9^th^ decile, 10^th^ decile), a scale of 44 recent life events (≤9^th^ decile, 10^th^ decile), chronic stress as measured by a family adversity index (≤9^th^ decile, 10^th^ decile).

### Statistical analysis

We first analysed the prevalence of high levels of anxiety symptoms according to key confounding variables that were determined in a theoretical model. We applied bivariate logistic regression analysis in order to evaluate the potential association of each independent covariate on the outcome. The strength of the association was measured using odds ratio (OR) and 95% confidence intervals (CIs). In order to allow flexibility of variable selection, those with a *P* value <0.20 on the bivariate analysis were incorporated in the multivariate model.

Each of the dietary pattern scores was categorized into tertiles and the lowest scoring group was considered the reference category. The independent effect of each dietary pattern was tested on the outcome, adjusting for all identified variables with *P* value <0.20 in the bivariate model plus total estimated dietary energy intake to control for different energy requirements. Additionally, on a final model we repeated the analysis adjusting for the total n-3 PUFA from seafood. These analyses aim to test whether the possible dietary pattern effect could occur in detriment of n-3 PUFA. Furthermore, we tested if there was an association with total n-3 PUFA intake from seafood, grouped into none; 0.1–0.4 g; 0.5–1.5 g; >1.5 g per week. The group with the highest intake was considered the reference. We performed logistic regression controlling for all confounders identified in the first stage, and for total energy intake. To test the association between the frequency of fish consumption and anxiety, we repeated the regressions with each of the three categories of fish intake provided in the food frequency questionnaire stratified as: never or rarely; once in 2 weeks or 1–3 times per week or more. Finally we analysed within each dietary pattern, for the women eating the different frequency of dark and oily fish, based on the above-mentioned categories, whether fish itself on top of a dietary pattern could modify the likelihood of anxiety occurrence.

We performed further analyses comparing those women included and those excluded in the current analysis due to any missing data using chi-square test for proportions for all confounding variables. All statistical analyses were conducted with the IBM Statistical Package for Social Science version 18.0.

## Results

### Study participants and confounding

**Table S1** shows the distribution among those included and those not included in the current analysis.

The variation in the prevalence of high levels of anxiety symptoms with the confounding variables is shown in **Table 1**. In bivariate analyses, greater odds of high levels of anxiety symptoms were observed among women <25 years, with lower education, unemployed, living in council-owned (public) housing; in households with more overcrowding, with 2 or more children, previous history of abortion and miscarriage, smokers, high levels of adverse life events in childhood and recently and high levels of chronic stressors as measured by the family adversity index.

**Table 1:** Prevalence of high levels of anxiety symptoms at 32 weeks of gestation according to selected variables.

			High levels of anxiety symptoms^1^			
Variable	Total *n*	%	*n*	%	Odds Ratio	*P* 2
					(95% CI)	
**Age**						
<25	2078	21.8	456	21.9	1.64 (1.45–1.86)	<0.01
≥25	7452	78.2	1089	14.6	1.00	
**Ethnicity**						
White	9344	98.0	1509	16.1	1.00	
Non-white	186	2.0	36	19.4	1.25 (0.86–1.80)	0.24
**Education**						
Low	2552	26.8	523	20.5	1.71 (1.49–1.96)	
Medium	3437	36.1	559	16.3	1.29 (1.13–1.47)	<0.01
High	3541	37.2	463	13.1	1.00	
**Work status**						
Employed	5936	62.3	832	14.0	1.00	
Unemployed	3594	37.7	713	19.8	1.52 (1.36–1.69)	<0.01
**Housing tenure**						
Mortgaged/owned	7463	78.3	1033	13.8	1.00	
Council	1100	11.5	287	26.1	2.20 (1.89–2.55)	<0.01
Other	967	10.1	225	23.3	1.89 (1.60–2.22)	
**Crowding (persons/room)**						
≤1	9029	94.7	1402	15.5	1.00	
>1	501	5.3	143	9.3	2.17 (1.77–2.66)	<0.01
**Parity**						
0	4294	45.1	641	14.9	0.94 (0.83–1.06)	0.94
1	3395	35.6	535	15.8	1.00	
≥2	1841	19.3	369	20.0	1.34 (1.16–1.55)	<0.01
**History of abortion**						
Yes	1268	13.3	275	21.7	1.52 (1.32–1.76)	<0.01
No	8262	86.7	1270	15.4	1.00	
**History of miscarriage**						
Yes	1932	20.3	367	19.0	1.28 (1.12–1.45)	<0.01
No	7598	79.7	1178	15.5	1.00	
**Smoking**						
Yes	2129	22.3	514	24.1	1.97 (1.74–2.21)	<0.01
No	7401	77.7	1031	13.9	1.00	
**Alcohol consumption**						
Yes	5281	55.4	855	16.2	1.00 (0.89–1.11)	0.94
No	4249	44.6	690	16.2	1.00	
**Childhood life events**						
1^st^–9^th^	8318	87.3	1141	13.7	1.00	
10^th^	1212	12.7	404	33.3	3.14 (2.75–3.60)	<0.01
**Recent life events**						
1^st^–9^th^	8480	89.0	1240	14.6	1.00	
10^th^	1050	11.0	305	29.0	2.39 (2.06–2.76)	<0.01
**Family adversity index**						
1^st^–9^th^	8928	93.7	1302	14.6	1.00	
10^th^	602	6.3	243	40.4	3.96 (3.33–4.71)	<0.01

ALSPAC cohort study (1991–1992) (*n* = 9,530).

1 Crown-Crisp Experiential Index score ≥9.

2 *P* values refer to binary logistic regression; variables with more than two categories refer to *P* for linear trend.

### Dietary patterns and anxiety

In multivariate logistic regression, both health-conscious and traditional patterns showed protective associations, which persisted even after n-3 PUFA from seafood had been taken into account. This was, in particular, due to women classified in the highest tertile of the health-conscious pattern (OR 0.77; 95% CI: 0.65–0.93) and to a trend for lower odds with high scores on the traditional pattern. Women classified in the middle (OR 1.24; 95% CI: 1.07–1.43) and the highest tertile (OR 1.25; 95% CI: 1.08–1.44) of the vegetarian pattern were more likely to have high levels of anxiety symptoms in comparison with those classified in the lowest tertile. The processed and confectionary dietary patterns did not show associations with high anxiety after multivariate adjustment (**Table 2**). Effect sizes were usually modified on the initial adjustment for social and maternal variables with much less change as a result of adjusting for n-3 PUFA intake from seafood.

**Table 2:** Unadjusted and adjusted associations between dietary patterns and high levels of anxiety symptoms at 32 weeks of gestation.

			**High levels of anxiety symptoms** 1				
					**Unadjusted**	**Adjusted^2^**	**Adjusted^3^**
**Dietary pattern**	**Total *n***	**%**	***n***	**%**	**Odds Ratio**	**Odds Ratio**	**Odds Ratio**
					**(95% CI)**	**(95% CI)**	**(95% CI)**
**Health-conscious**							
1^st^ tertile	3007	31.6	607	20.2	1.00	1.00	1.00
2^nd^ tertile	3206	33.6	538	16.8	0.80 (0.70–0.91)	0.99 (0.85–1.14)	1.03 (0.89–1.19)
3^rd^ tertile	3317	34.8	400	12.1	0.54 (0.47–0.62)	0.71 (0.60–0.84)	0.77 (0.65–0.93)
* P for trend*					<0.01	<0.01	<0.01
**Traditional**							
1^st^ tertile	3176	33.3	563	17.7	1.00	1.00	1.00
2^nd^ tertile	3225	33.8	494	15.3	0.84 (0.74–0.96)	0.87 (0.76–1.00)	0.88 (0.76–1.01)
3^rd^ tertile	3129	32.8	488	15.6	0.86 (0.75–0.98)	0.83 (0.72–0.95)	0.84 (0.73–0.97)
* P for trend*					<0.01	<0.01	0.02
**Processed**							
1^st^ tertile	3195	33.5	458	14.3	1.00	1.00	1.00
2^nd^ tertile	3263	34.2	511	15.7	1.11 (0.97–1.27)	1.02 (0.88–1.18)	1.02 (0.88–1.18)
3^rd^ tertile	3072	32.2	576	18.8	1.38 (1.21–1.58)	1.03 (0.88–1.21)	1.03 (0.88–1.21)
* P for trend*					<0.01	0.67	0.63
**Confectionery**							
1^st^ tertile	3067	32.2	487	15.9	1.00	1.00	1.00
2^nd^ tertile	3226	33.9	469	14.5	0.90 (0.78–1.03)	0.96 (0.83–1.11)	0.93 (0.81–1.08)
3^rd^ tertile	3237	34.0	589	18.2	1.18 (1.03–1.34)	1.24 (1.06–1.45)	1.16 (0.99–1.37)
* P for trend*					0.01	<0.01	0.06
**Vegetarian**							
1^st^ tertile	3275	34.4	430	13.1	1.00	1.00	1.00
2^nd^ tertile	3189	33.5	550	17.2	1.37 (1.20–1.58)	1.25 (1.08–1.45)	1.24 (1.07–1.43)
3^rd^ tertile	3066	32.2	565	18.4	1.49 (1.30–1.71)	1.25 (1.08–1.45)	1.25 (1.08–1.44)
* P for trend*					<0.01	<0.01	<0.01

ALSPAC cohort study (1991–1992) (*n* = 9,530).

1 Crown-Crisp Experimental Index score ≥9.

2 Adjusted for maternal estimated energy intake, age, education, work status, housing, crowding, parity, past history of abortion, past history of miscarriage, smoking habit, childhood life events, recent life events, and family adversity index.

3 Additionally adjusted for maternal estimated intake of n-3 PUFA from seafood.

### Seafood exposure and anxiety

**Figure 1:**
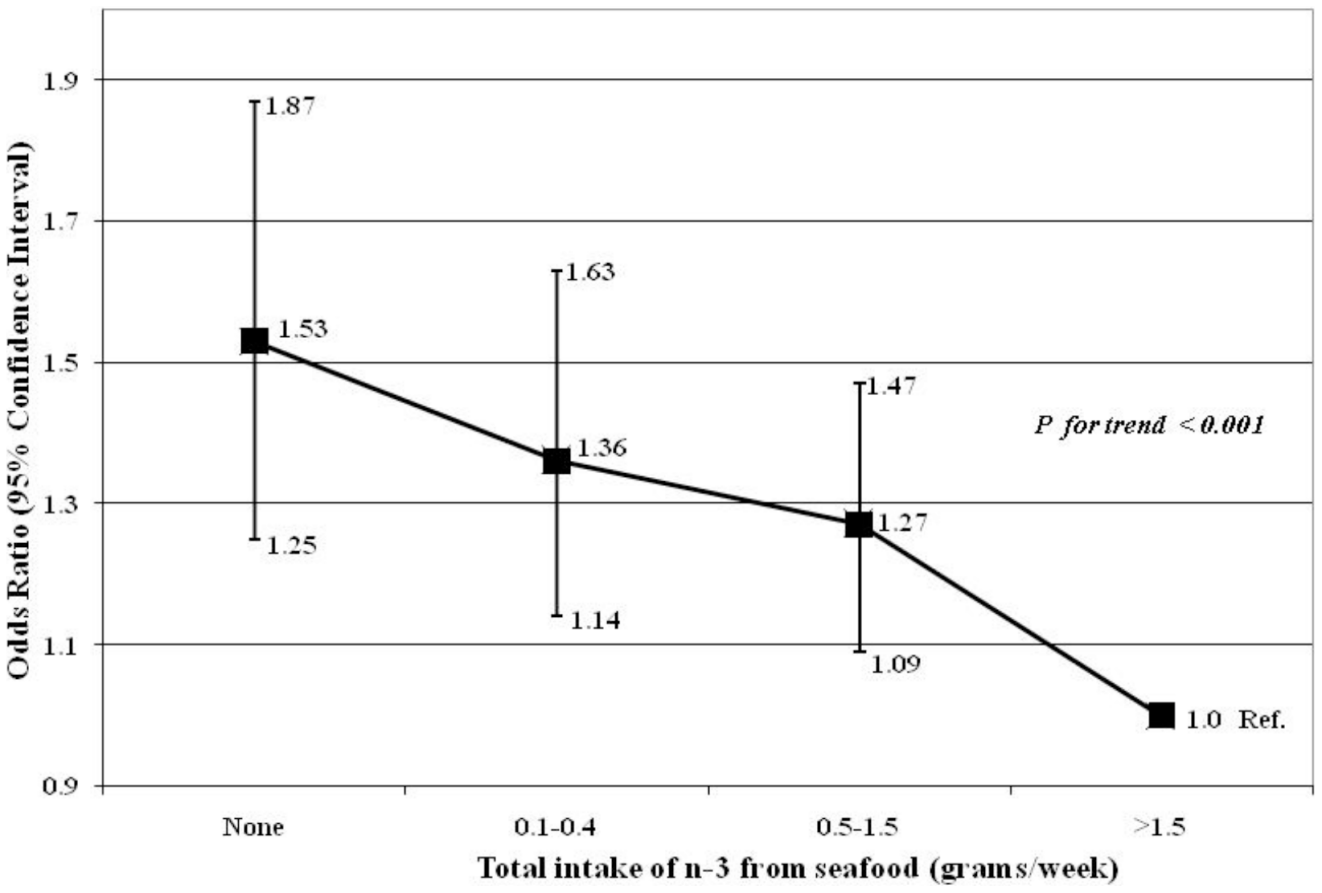
Data show odds ratios (OR) and 95% confidence intervals (CI) for high anxiety symptoms measured at 32 weeks of gestation. Values are as follows for frequency of intake of n-3 from seafood (grams/week): None: OR  = 1.53, 95% CI: 1.25–1.87, n = 239/1140; 0.1–0.4 g: OR  = 1.36, 95% CI: 1.14–1.63, n = 333/1838; 0.5–1.5 g: OR  = 1.27, 95% CI: 1.09–1.47, n = 657/4072; N-3 PUFA intake >1.5 g was the reference category (n = 316/2480). All analysis were adjusted for maternal estimated energy intake, age, education, work status, housing tenure, crowding, parity, previous history of abortion, previous history of miscarriage, smoking habit, childhood life events, recent life events, and family adversity index.

**Figure 2:**
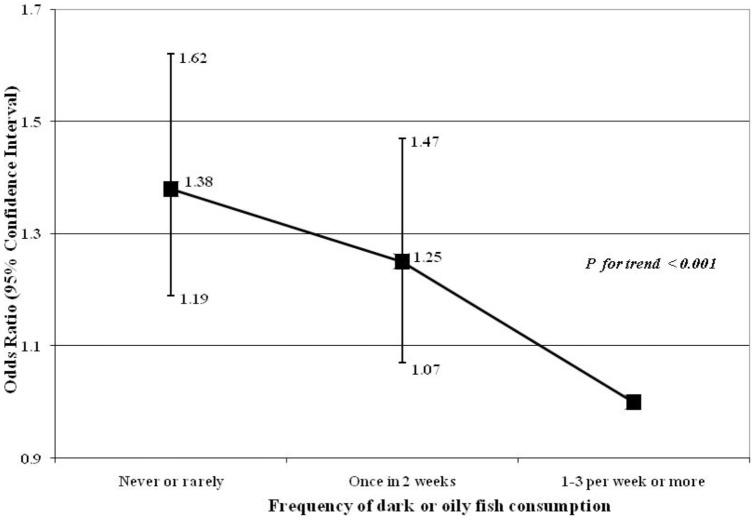
Data show odds ratios (OR) and 95% confidence intervals (CI) for high anxiety symptoms measured at 32 weeks of gestation. Values are as follows for frequency of dark or oily fish consumption: Never or rarely: OR  = 1.38, 95% CI: 1.19–1.62, n = 739/3896; once in 2 weeks: OR  = 1.25, 95% CI: 1.07–1.47, n = 508/3240; 1–3 per week or more was the reference category (n = 298/2394). All analysis were adjusted for maternal estimated energy intake, age, education, work status, housing tenure, crowding, parity, previous history of abortion, previous history of miscarriage, smoking habit, childhood life events, recent life events, and family adversity index.

Multivariable logistic regression using intake of n-3 PUFA from seafood as the exposure and anxiety symptoms as the outcome showed the adjusted odds for high levels of anxiety symptoms for those with no n-3 PUFA intake from seafood at all (OR 1.53; 95% CI: 1.25–1.87) when compared with those with intake of >1.5 grams/week. The likelihood of high anxiety symptoms was also observed for those with n-3 PUFA intake between 0.1–0.4 grams/week (OR 1.36; 95% CI: 1.14–1.63) and for those with intake between 0.5–1.5****grams/week (OR 1.27; 95% CI: 1.09–1.47) (**Figure 1**). The likelihood of high anxiety was observed for those who reported never or rarely consuming dark or oily fish (OR 1.38; 95% CI: 1.19–1.62) and once in 2 weeks (OR 1.25; 95% CI: 1.07–1.47), compared to those who reported intakes of 1 to 3 times per week or more (**Figure 2**).

The frequency of dark and oily fish intake among women inside each dietary pattern revealed a significant increase in the odds for high anxiety symptoms when those with intake of 1–3 times a week or more were compared with those that consumed fish rarely or never. The observed odds were as follows: health-conscious (OR 1.57, 95% CI: 1.19–2.08); traditional (OR 1.64, 95% CI: 1.26–2.16); confectionary (OR 1.47, 95% CI: 1.13–1.92) and vegetarian (OR 1.39, 95% CI: 1.07–1.80). The results were significant among all dietary patterns, except for the processed one (**Table S2**).

## Discussion

The main finding of the present study is that women that scored in the highest tertile of health-conscious or traditional dietary patterns were 23% and 16% less likely to have high levels of anxiety symptoms at the 32 weeks of pregnancy, respectively when compared with those women classified in the lowest tertile of such patterns. Women that scored in the highest tertile of a vegetarian pattern had 25% greater likelihood for high levels of anxiety symptoms, when compared with women classified in the lowest tertile. In addition, women with no intake of n-3 PUFA from seafood or that reported never or rarely for frequency of dark or oily fish consumption, had respectively 53% and 38% greater likelihood of high levels of anxiety, when compared with women with n-3 PUFA intake from seafood >1.5 grams/week, or who consumed dark or oily fish 1–3 times a week or more. It was interesting to note that even for women who adhere to a vegetarian pattern, the likelihood of anxiety was 43% higher for those that ate dark and oily fish rarely or never in comparison with those that ate 1–3 times a week or more. We observed a similar behaviour for all other patterns, except for the processed one. The associations remained significant even after adjustment for potential confounders. The dietary patterns analysis results were robust to adjustment for n-3 PUFA intake from seafood. Neither processed nor confectionary patterns were associated with high anxiety symptoms in the fully adjusted analysis, although the later one presented borderline association (*P* = 0.06). To our knowledge this is the first study describing a relationship between dietary patterns and high levels of anxiety symptoms during pregnancy, and the first to provide evidence of low n-3 PUFA intake and low fish consumption being associated with high anxiety symptoms or anxiety-related conditions.

The strengths of the study include the large sample size; 9,530 pregnant women and also the ability to perform fully adjusted regression models with a large number of confounders. Furthermore, the principle components analysis used to obtain the dietary patterns is currently the most recommended procedure [25]. The five dietary components defined for the sample of pregnant women of ALSPAC were chosen to best describe the dietary patterns, explaining 32.7% of the variance and has been used in another ALSPAC investigation on diet-disease [27]. The sample size included in the present study represents a substantial proportion of the original sample (66%) and thus unlikely to compromise the external validity.

The main limitation of the present study relates to possible reverse causality. Both exposure and outcome were assessed at the same time point (32 weeks of gestation), which may not reflect the entirety of pregnancy. However, the study of Crozier et al. (2009) [28] showed limited change in fish consumption during pregnancy, with no difference in the weekly frequency from before to late in pregnancy. The multiple food items and choices in a food frequency questionnaire may also result in an overestimation of the intakes of energy and some specific nutrients – such as n-3 PUFA. Also the retrospective nature of the questionnaire may introduce some memory bias, when compared with the prospective and ‘gold standard’ – weighed dietary records. Another potential limitation is the restriction of the estimate of n-3 PUFA to seafood. Although fish is a main contributor to dietary long chain essential n-3 PUFA, these are also present in small amounts in a few other foods (such as meat). Although we have not used a biological marker of n-3 PUFA intake in the present work, previous ALSPAC publications have confirmed strong correlations between maternal intake of oily fish and maternal n-3 PUFA concentrations in red blood cell membranes [29], [30].

Our findings are consistent with previous investigations of dietary patterns and anxiety among adult women, and add new evidence regarding high anxiety during pregnancy. In a cross-sectional analyses of 1,046 Australian adult women followed up in a longitudinal cohort study, Jacka et al. (2010) [31] demonstrated a 33% protective effect of a “traditional” dietary pattern characterized by vegetables, fruit, meat, fish, and whole grains on anxiety. In a larger cohort study – the Hordaland Health Study, diet of 3,254 women was analysed [32]. Those women that scored highly on a “healthy” dietary pattern (characterized by vegetable, salad, fruit, cereal, fish, wine and non-processed meat) were less likely to present a high anxiety score, whereas this was more likely for women scoring highly on the “western” pattern (characterized by processed meat, refined carbohydrates and industrialized food based on vegetable oil). There is limited literature exploring dietary patterns and psychological features in pregnancy, and previous studies have focused on depressive symptoms [18], [33]. In a sample of 529 pregnant women from a population-based study in Crete, Greece, higher scores on a dietary pattern during pregnancy based on vegetables, fruit, pulses, nuts, dairy products, fish, seafood, and olive oil (defined as “health-conscious”) showed a 50% protective effect against high levels of postpartum depressive symptoms [18].

Our findings of a likelihood of low anxiety symptoms among women with intakes of >1.5 grams/week of n-3 PUFA are in line with previous clinical investigations of PUFA and anxiety disorders. Clinical investigations have provided strong evidence that consumption of long chain n-3 PUFA presents significant anxiolytic benefits. A randomized clinical trial among medical students without a diagnosis of anxiety disorder reported a 20% reduction in anxiety symptoms after 2.5g/day of n-3 PUFA supplementation during a period of stress compared to placebo [34]. Moreover, clinical investigation of PUFA concentrations in red blood cells among 27 untreated, non-depressed subjects with social anxiety disorder showed a lower concentration of most essential n-3 PUFA (18:3n-3 by 32%; 20:5n-3 by 36% and 22:6n-3 by 18%) compared to control subjects [35]. Although no inferences can be drawn regarding causation, such data suggest that anxiety might be associated with either decreased n-3 PUFA intakes or decreased uptake of n-3 PUFA into cell membranes [36].

We observed a protective effect of high scores on traditional and health-conscious dietary patterns and an increased likelihood of high anxiety symptoms with the vegetarian (or vegetable protein) pattern. The main difference between the first two dietary patterns and the vegetarian one is the greater likelihood of animal sources – meat and fish – in the traditional and health-conscious patterns, respectively. It is unlikely that women who were anxious about their pregnancy turned to vegetarianism since in ALSPAC only 4% of vegetarians had been eating in this way for less than one year. In the modern diet, meat is the richest source of vitamin B12, and fish is the main source of long-chain essential n-3 PUFA, especially docosahexaenoic acid (DHA; 22:6n-3). These nutrients are necessary for optimal neurological function and signs of neurocognitive impairment are reflected in mood changes [37]. Although a vegetarian diet is often claimed to be healthy because of the low concentrations of saturated fat and cholesterol and the high proportion of dietary fiber [38], [39], some investigations have reported that individuals who adhere to a vegetarian diet present lower tissue concentrations of long chain n-3 PUFA [40] and vitamin B12 [41], [42]. Additionally, nutritional requirements of these nutrients are increased during pregnancy due to the demands of the fetus, which may then result in maternal deficiency if not balanced by nutritional supplements [43]–[45]. In particular in regard to essential fatty acids, the main source of n-3 PUFA in vegetarian diets is α-linolenic acid (ALA; 18:3n-3), which is the precursor of eicosapentaenoic acid (EPA; 20:5n-3) and DHA. However, vegetarian diets may supply more linoleic acid (LA; 18:2n-6) than omnivorous diets [46] and a high ratio of LA:ALA suppresses the elongation of n-3 PUFA forms and favors the production of docosapentaenoic acid (DPA; 22:5n-6), which replaces DHA in neural tissues [36].

The results further suggest an independent relationship between low n-3 PUFA intake and fish consumption and anxiety. Although no inference can be drawn regarding causation such data do suggest that a lower intake of n-3 PUFA or low dark and oily fish consumption is associated with higher levels of anxiety symptoms. Pregnancy is associated with a significant decrease of n-3 PUFAs in tissues due to high transference to the fetus, which may be related to a decrease in uptake of n-3 PUFA into cell membranes consequent upon fetal demands [47], [48]. A clinical investigation has shown that subjects with either lower n-3 PUFA serum levels or a high n-6/n-3 ratio, when exposed to psychological stress, had an increase in proinflammatory cytokines [49]. These cytokines promote secretion of corticotrophin-releasing hormone (CRH), a primary gateway to hormonal stress responses [50]. CRH also stimulates the amygdala, a key brain region for fear and anxiety. Hibbeln et al. (2004) [51] have reported that higher concentrations of DHA in serum are associated with lower CRH in spinal fluid from violent offenders. Gestational anxiety is mainly related to concerns in regard to the fetus and birth [1]. Thus, a diet low in n-3 PUFA – especially of oily fish – might indicate lower n-3 tissue concentrations and represent an increased risk for a physiological response with anxiogenic effect when exposed to stress.

The present study provides evidence of a significant relationship between dietary patterns and symptoms of anxiety in pregnancy, and suggests that dietary interventions might possibly be used to prevent or reduce high anxiety symptoms during pregnancy. From a clinical perspective, the current findings indicate that dietary counselling during prenatal care with a focus on healthy habits and increased n-3 PUFA intake from seafood consumption may be beneficial to women with high anxiety during pregnancy. However, appropriately designed clinical trials need to be conducted to evaluate the efficacy of such interventions.

## Supporting Information

Table S1 **Distribution of potential confounders among those women included (** ***n*** **= 9,530) and those not included (** ***n*** **= 5,011) in the current study.**
(PDF)

Table S2 **Investigating the frequency of fish consumption within women in the third tertile of adherence to each dietary pattern, a significant increase in the odds for high anxiety symptoms among those women with lower frequency of fish consumption is observed.** This trend is found in all dietary patterns, except the processed one.
(PDF)
