# Supplementary material for: Dietary Patterns, n-3 Fatty Acids Intake from Seafood and High Levels of Anxiety Symptoms during Pregnancy: Findings from the Avon Longitudinal Study of Parents and Children
Source: PLoS One. 2013 Jul 12;8(7):e67671. doi: 10.1371/journal.pone.0067671 (PMC3710017; doi:10.1371/journal.pone.0067671)
Supplement: Table S1 — Distribution of potential confounders among those women included ( n = 9,530) and those not included ( n = 5,011) in the current study. (PDF) [file pone.0067671.s001.pdf]

Table S1. Frequency distribution of main investigated variables between those who were included in the analyses and those who not. ALSPAC cohort study (1991-1992)

Variable	Included in the analyses		Excluded by missing information		*P* value[1]
	n	%	*n*	%	
Age					
<25	2078	21.8	1202	33.5	<0.001
≥25	7452	78.2	2385	66.5	
Ethnicity					
White	9344	98.0	2416	95.0	<0.001
Non-white	186	2.0	128	5.0	
Education					
Low	2552	26.8	1104	41.9	
Medium	3437	36.1	774	29.4	<0.001
High	3541	37.2	757	28.7	
Work status					
Employed	5936	62.3	1032	47.1	<0.001
Unemployed	3594	37.7	1159	52.9	
Housing tenure					
Mortgaged/owned	7463	78.3	2143	59.9	
Council	1100	11.5	784	21.9	<0.001
Other	967	10.1	649	18.1	
Crowding (persons/room)					
≤1	9029	94.7	2957	88.4	<0.001
>1	501	5.3	388	11.6	

Cont.

Variable	Included in the analyses		Excluded by missing information		*P* value[1]
	n	%	*n*	%	
Parity					
0	4294	45.1	1431	44.6	
1	3395	35.6	1037	32.4	<0.001
≥2	1841	19.3	737	23.0	
Previous history of abortion					
Yes	1268	13.3	592	18.4	<0.001
No	8262	86.7	2631	81.6	
Previous history of miscarriage					
Yes	1932	20.3	811	24.8	<0.001
No	7598	79.7	2456	75.2	
Smoking					
Yes	2129	22.3	1116	32.5	<0.001
No	7401	77.7	2318	67.5	
Alcohol consumption					
Yes	5281	55.4	1716	52.4	<0.001
No	4249	44.6	1561	47.6	
Childhood life events (decile)					
1st - 9th	8318	87.3	1895	82.9	<0.001
10th	1212	12.7	392	17.1	
Recent life events (decile)					
1st - 9th	8480	89.0	2232	86.3	<0.001
10th	1050	11.0	354	13.7	
Family adversity index (decile)					
1st - 9th	8928	93.7	3096	90.4	<0.001
10th	602	6.3	330	9.6	

[1]*P* value refers to chi-square test for comparisons of sample distribution of investigated variables.
