# Supplementary material for: Dietary Patterns, n-3 Fatty Acids Intake from Seafood and High Levels of Anxiety Symptoms during Pregnancy: Findings from the Avon Longitudinal Study of Parents and Children
Source: PLoS One. 2013 Jul 12;8(7):e67671. doi: 10.1371/journal.pone.0067671 (PMC3710017; doi:10.1371/journal.pone.0067671)
Supplement: Table S2 — Investigating the frequency of fish consumption within women in the third tertile of adherence to each dietary pattern, a significant increase in the odds for high anxiety symptoms among those women with lower frequency of fish consumption is observed. This trend is found in all dietary patterns, except the processed one. (PDF) [file pone.0067671.s002.pdf]

Table S2. Odds ratio (95% Confidence Interval) for high levels of anxiety symptoms among women classified in the third tertile of each dietary pattern according to the frequency of dark and oily fish consumption. ALSPAC cohort study (1991-1992) (n = 9,530)

Dietary pattern	Total *n*	High levels of anxiety symptoms[1] Frequency of dark and oily fish consumption		
		Never or rarely Odds Ratio (95% CI)[2]	Once in two weeks Odds Ratio (95% CI)[2]	1-3 times per week or more Odds Ratio (95% CI)[2]
**Health-conscious**	3,317	1.57 (1.19-2.08) [*n* = 688]	1.30 (1.01-1.67) [*n* = 1,175]	1.0 [*n* = 1,454]
P value[3]		0.002	0.043	
**Traditional**	3,129	1.64 (1.26-2.16) [*n* = 1,135]	1.46 (1.11-1.91) [*n* = 1,115]	1.0 [*n* = 879]
P value[3]		<0.001	0.006	
**Processed**	3,072	1.02 (0.79-1.31) [*n* = 1,414]	1.08 (0.83-1.42) [*n* = 896]	1.0 [*n* = 762]
P value[3]		0.872	0.541	0.799
**Confectionary**	3,237	1.47 (1.13-1.92) [*n* = 1,429]	1.29 (0.98-1.70) [*n* = 1,089]	1.0 [*n* = 719]
P value[3]		0.004	0.068	
**Vegetarian**	3,066	1.39 (1.07-1.80) [*n* = 1,307]	1.25 (0.96-1.64) [*n* = 916]	1.0 [*n* = 843]
P value[3]		0.013	0.101	

[1]Crown-Crisp Experiential Index score ≥ 9.

[2]Adjusted for maternal estimated energy intake, age, education, work status, housing, crowding, parity, past history of abortion, past history of miscarriage, smoking habit, childhood life events, recent life events, and family adversity index.

[3]*P* value refers to binary logistic regression.
